# Goethite Mineral Dissolution to Probe the Chemistry of Radiolytic Water in Liquid‐Phase Transmission Electron Microscopy

**DOI:** 10.1002/advs.202301904

**Published:** 2023-07-13

**Authors:** Thaïs Couasnon, Birk Fritsch, Michael P. M. Jank, Roberts Blukis, Andreas Hutzler, Liane G. Benning

**Affiliations:** ^1^ GFZ German Research Center for Geosciences Telegrafenberg 14473 Potsdam Germany; ^2^ Department of Electrical, Electronic, and Communication Engineering Electron Devices Friedrich‐Alexander‐Universität Erlangen‐Nürnberg 91058 Erlangen Germany; ^3^ Department of Materials Science and Engineering Institute of Micro‐ and Nanostructure Research (IMN) and Center for Nanoanalysis and Electron Microscopy (CENEM) Friedrich‐Alexander‐Universität Erlangen‐Nürnberg 91058 Erlangen Germany; ^4^ Forschungszentrum Jülich GmbH Helmholtz Institute Erlangen‐Nürnberg for Renewable Energy (IEK‐11) 91058 Erlangen Germany; ^5^ Fraunhofer Institute for Integrated Systems and Device Technology IISB Schottkystr. 10 91058 Erlangen Germany; ^6^ Leibniz‐Institut für Kristallzüchtung Max‐Born Str. 2 12489 Berlin Germany; ^7^ Department of Earth Sciences Freie Universität Berlin 12249 Berlin Germany

**Keywords:** dissolutions, electron beam effects, goethite, liquid‐phase transition electron microscopy, water radiolysis

## Abstract

Liquid‐Phase Transmission Electron Microscopy (LP‐TEM) enables in situ observations of the dynamic behavior of materials in liquids at high spatial and temporal resolution. During LP‐TEM, incident electrons decompose water molecules into highly reactive species. Consequently, the chemistry of the irradiated aqueous solution is strongly altered, impacting the reactions to be observed. However, the short lifetime of these reactive species prevent their direct study. Here, the morphological changes of goethite during its dissolution are used as a marker system to evaluate the influence of radiation on the changes in solution chemistry. At low electron flux density, the morphological changes are equivalent to those observed under bulk acidic conditions, but the rate of dissolution is higher. On the contrary, at higher electron fluxes, the morphological evolution does not correspond to a unique acidic dissolution process. Combined with kinetic simulations of the steady state concentrations of generated reactive species in the aqueous medium, the results provide a unique insight into the redox and acidity interplay during radiation induced chemical changes in LP‐TEM. The results not only reveal beam‐induced radiation chemistry via a nanoparticle indicator, but also open up new perspectives in the study of the dissolution process in industrial or natural settings.

## Graphical abstract

In situ Liquid‐Phase Transmission Electron Microscopy observations of the morphological changes of goethite nanoparticles during its dissolution are used to evaluate the influence of radiation on changes in solution chemistry. The results not only reveal beam‐induced radiation chemistry via a nanoparticulate indicator, but also open up new perspectives in the study of dissolution processes in industrial and natural settings.

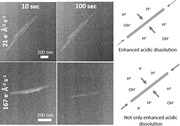

## 1 Introduction

The dissolution of (nano)materials is of great interest to investigate to a wide range of research fields, including climate and atmospheric CO_2_ storage associated with rock erosion,^[^ ^1^, ^2^, ^3^ ^]^ agricultural nutrient release strategies,^[^ ^4^ ^]^ bioweathering in ecology,^[^ ^5^ ^]^ energy storage in batteries,^[^ ^6^ ^]^ and corrosion science^[^ ^7^ ^]^ involving biomaterials,^[^ ^8^ ^]^ but also in glass industry.^[^ ^9^ ^]^ So far, in many of these fields, an in situ and real time study of the processes involved has been prevented by technical limitations, yet, LP‐TEM is a promising accelerated testing option. Transmission electron microscopy (TEM) facilitates visualization and analyses of matter at the nanoscale and helps to unravel the composition and structure of solids. In conventional TEM, the samples are analyzed in a dried state to withstand the high vacuum inside the TEM column. Over the past years, LP‐TEM has been developed to overcome the limitations of conventional TEM failing at addressing nanoscopic dynamic changes in materials in the presence of a solvent, and under atmospheric pressure conditions.^[^ ^10^ ^]^

LP‐TEM is a powerful technique that has already shown great capabilities to image nanoscale biological systems, such as viruses,^[^ ^11^ ^]^ as well as highly reactive inorganic materials, such as in lithium‐ and sodium‐ion battery processes.^[^ ^12^ ^]^ In addition, LP‐TEM has also been exploited to investigate how nanoparticles form in various liquid environments, challenging the classical representation of nucleation and growth. Among others, LP‐TEM revealed the presence of condensed atomic clusters during Pd and Au crystallization,^[^ ^13^ ^]^ and could be used to trigger on‐demand nucleation and growth of calcite from precursor nanodroplets.^[^ ^14^ ^]^ LP‐TEM is therefore a promising tool to answer fundamental environmental and geochemical questions regarding the transport and bioavailability of elements in natural systems.

Many LP‐TEM developments are performed in hermetically sealed cells containing the sample and its native surrounding liquid medium. During imaging, a larger electron flux improves the image quality with an increased signal to noise ratio. This occurs at the cost of locally altering the solution chemistry by inducing radiolysis of the liquid phase. Similar to other kinds of ionizing radiation, the interaction of fast electrons with water causes the formation of reactive species, such as hydrated electrons e_h_^−^, hydrogen radicals H^•^, or hydroxyl radicals OH^•^.^[^ ^15^, ^16^ ^]^ These species diffuse within the surrounding liquid and will invariably induce subsequent chemical reactions. Continual irradiation, quickly results in a steady state concentration of reactive species in the irradiated imaging area.^[^ ^17^ ^]^

The generation of reactive species influences the chemistry within the solution and can be tailored to a certain extent.^[^ ^18^, ^19^ ^]^ For example, radiolysis of aqueous precursor solutions under electron irradiation has been exploited as a reaction stimulus for nanoparticle formation via reduction^[^ ^20^, ^21^, ^22^ ^]^ or particle dissolution via oxidation.^[^ ^23^, ^24^, ^25^, ^26^ ^]^ The stimulus also was shown to trigger reversible redox‐mediated transformation,^[^ ^9^, ^17^, ^18^ ^]^ as well as anisotropic changes in silica nanoparticle shapes.^[^ ^29^ ^]^ To better understand the influence and distribution of these reactive species, kinetic modeling has been used to predict their concentrations.^[^ ^17^, ^19^, ^28^, ^30^, ^31^ ^]^ In addition, Schneider et al. introduced a solid foundation of pure water radiolysis simulations to anticipate changes in the aqueous chemistry in LP‐TEM experiments,^[^ ^17^ ^]^ and quantitative implications on redox processes have been recently enabled by an approach utilizing gold nanoparticle evolution and an extension of the reaction model beyond pure water.^[^ ^9^ ^]^

Furthermore, it was suggested that the electron beam induces an acidification of the irradiated pure water.^[^ ^17^ ^]^ However, in the presence of dissolved aqueous ion precursors, the same electron irradiation can also trigger alkalinization of the surrounding aqueous medium.^[^ ^22^, ^32^ ^]^ This is related to the simultaneous formation of both, H^+^ and OH^−^ under irradiation. OH^−^ concentration evolution was neglected in previous studies, but was recently shown to disqualify pH as a meaningful descriptor of acidity in LP‐TEM.^[^ ^27^ ^]^ Hence, an alternative concept to capture acidity in LP‐TEM was proposed, namely the radiolytic acidity π* and radiolytic ion product *K*_w_*.^[^ ^27^ ^]^ However, this concept still lacks the required combination of systematic experimental evidence with appropriate modeling of radiation chemistry.

Therefore, one of the main current challenges in LP‐TEM is to unravel and disentangle the reactivity of solutions (acidity and redox conditions) under electron irradiation. Since the analysis of changes in bulk aqueous chemical compositions inside a LP‐TEM is not accessible, another proxy sensitive to oxidoreduction or acidity has to be used. To enable detailed chemical investigations performed under electron irradiation, such a proxy has to be based on a distinct material transformation that can be exploited as an indicator reaction (crystalline structure, particle morphology, etc.) in combination with suitable kinetic modeling.

To address this issue, we investigated in this study the in situ dissolution of redox active metal oxides and in particular the dissolution of a common iron oxyhydroxide, goethite. Similar materials play a key role in many natural and industrial processes, including catalysts,^[^ ^33^, ^34^, ^35^ ^]^ corrosion of metal oxides and steel^[^ ^36^ ^]^ as well as elemental biogeochemical cycling in natural environments.^[^ ^37^, ^38^, ^39^ ^]^ Thus, the simultaneous quantification and identification of dissolution locations in iron oxyhydroxide is of prime importance.^[^ ^40^ ^]^ Furthermore, LP‐TEM research has up to now mainly focused on metals,^[^ ^13^, ^41^, ^42^ ^]^ simple metal oxides,^[^ ^22^, ^23^ ^]^ and carbonates,^[^ ^43^ ^]^ however, iron oxides and goethite in particular are great candidates to tackle dissolution processes occurring in more complex materials, such as redox active metal oxides.

Goethite (α‐FeOOH) is a ferric oxyhydroxide phase that dissolves in bulk experiments under acidic or reductive conditions, or in the presence of chelating agents, but not at mild basic pH.^[^ ^44^, ^45^ ^]^ Goethite is acicular in shape and under acidic solutions, the edges of goethite nanoparticles (i.e., the (001) and (010) crystal faces) dissolve remarkably faster than the (100) facets, resulting in a pronounced anisotropic dissolution.^[^ ^46^ ^]^ On the other hand, Joshi et al. demonstrated distinct anisotropic morphological changes in goethite crystals due to the preferential etching of tips (i.e., the (210) and (234) crystal faces) following Fe^2+^‐catalyzed reductive dissolution,^[^ ^47^ ^]^ suggesting that goethite is a good candidate to monitor anisotropic dissolution also in reducing media.

Thus, we use the anisotropic dissolution behavior of goethite crystals to probe and monitor changes in the chemistry of the water medium in a liquid cell during electron irradiation. We compared the relative changes in lengths and widths of goethite crystals exposed to bulk reductive, basic, and acidic conditions to the relative changes obtained for crystals that were exposed to in situ electron‐irradiated aqueous solutions inside the LP‐TEM. To achieve this and to ensure a reliable comparison of the dissolution behavior, the same starting material was investigated to remove inconsistencies from variations in synthesis methods or the presence of structural defects. The experimental observations were compared to simulations of radiation effects in aqueous media containing dissolved iron as aqueous ions, utilizing the simulation tool developed by Fritsch et al.^[^ ^9^ ^]^

With these results we provide more quantitative insights and a better prediction capability of the chemical nature of water surrounding the solid system of interest during an LP‐TEM experiment. This is of utmost importance to understand the mechanisms involved in mineral nucleation, growth and dissolution and, thus, the elemental mobility in the environment, as well as the engineered processes and biological structures investigated in their native aqueous environments.

## 2 Results and Discussion

### 2.1 In Situ Liquid Cell Goethite Dissolution

**Figure 1:**
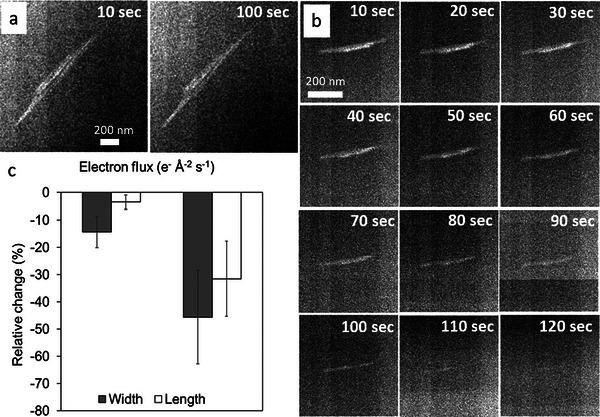
In situ monitoring of goethite dissolution. Contrast‐adjusted time series of goethite particles irradiated with an electron flux density of a) 21 e^−^ Å^−2^ s^−1^ and b) 167 e^−^ Å^−2^ s^−1^. c) Relative change in width and length of nanoparticles exposed 100 s in the LP‐STEM at an electron dose of 21 and 167 e^−^ Å^−2^ s^−1^.

The synthetic goethite particles used have a typical acicular crystal habit (**Figure** **1**). In the liquid cell, goethite nanocrystal dissolution reactions were monitored by sequential imaging at two different electron flux densities (21 and 167 e^−^ Å^−2^ s^−1^). Since the morphology did not change notably during an irradiation with 21 e^−^ Å^−2^ s^−1^, only the initial and final images are presented in Figure 1a (see also Video S1, Supporting Information). In contrast, the time‐resolved morphological evolution of nanocrystals irradiated with an electron flux density of 167 e^−^ Å^−2^ s^−1^ was much more substantial (Figure 1b and Video S2, Supporting Information). The results showed that goethite crystals were dissolved within 120 s at higher electron flux density. Therefore, the iron was transferred from the mineral structure to the surrounding aqueous medium.

Throughout the monitored reactions, no precipitation was observed either inside or outside of the irradiated window, suggesting that during goethite dissolution the iron was transferred from the mineral structure into the surrounding aqueous medium. In the present study, the Pourbaix diagram (Figure S2, Supporting Information) showed that iron‐based minerals will precipitate at pH > 7. Considering that our experiments started at a pH of 5.5, and combined with the absence of newly formed precipitation in our system, the results question the hypothesis of an increase in basicity of the surrounding medium during electron irradiation. “Although thermodynamics may be altered in a confined and irradiated medium,^[^ ^19^, ^48^, ^49^, ^50^ ^]^ Pourbaix Eh‐pH diagrams have been previously used to explain mineral precipitation in LP‐TEM.”

However, contrary to our experiments involving iron species, some authors suggested that precipitation occurred with a pH increase. First, Abellan et al. suggested that in the presence of dissolved cerium, electron beam interaction with the aqueous solution induces a pH increase leading to the formation of small irregular Ce(OH)_3_ nanoparticles.^[^ ^22^ ^]^ Another study also combined Pourbaix diagram analysis and in situ LP‐TEM observations and reported the increase in pH in an electron irradiated solution lead to the transformation of HCO_3_^−^ to CO_3_^2−^, and subsequent local increase in supersaturation inducing the precipitation of CoCO_3_.^[^ ^32^ ^]^ Therefore, the results in the present study, demonstrate that the irradiation‐mediated change in acidity or basicity strongly depends on the solutes themselves.

The transformation of another iron oxyhydroxide, akaganeite (β‐FeOOH), was studied in situ at the microscopic scale.^[^ ^43^ ^]^ Interestingly, the authors showed that aggregated akaganeite nanorods assembled and transformed into thermodynamically stable phase hematite (α‐Fe_2_O_3_). Their results hence followed thermodynamic predictions and differ from the present study where α‐FeOOH nanoparticles dissolve. In their experiment, the authors were not aiming at quantifying the transformation process, therefore there is unfortunately no further insight in the electron fluxes used to image the transformation process. Besides, our results also stress the importance of monitoring the LP‐TEM settings (imaging parameters, electron flux, and liquid cell thickness) to further understand the processes occurring in situ.

To gain further insights into the two possible dissolution mechanisms (i.e., reductive and acidic dissolution), we analyzed the time‐resolved relative changes in dimension as the shape anisotropy was preserved. Figure S3 (Supporting Information) shows the width and length dimension changes from four particles in two independent, but equivalent experiments.

Based on this data we quantified the structural evolution (relative change in width and length) as a function of time using the following expression:

(1) $$\mathrm{Relative}\;\mathrm{change}\;=\;\frac{\left(d_{f}-d_{0}\right)}{d_{0}}$$

where *d*_f_ are the dimensions (width or length) after exposure and *d*_0_ the dimensions of the pristine particle (i.e., the first image of the LP‐TEM experiment). A negative relative change describes a decrease in widths and lengths as compared to the initial dimensions of the particle. On the contrary, a positive relative change reveals that the final measured dimensions are larger than the initial ones.

The first important observation was that no dimensional change in width and length could be measured when images of goethite nanocrystals equilibrated inside the liquid of the LP‐TEM cell were acquired before and after 100 s, but without electron beam irradiation (electron beam blanked during 100 s). Therefore, without electron irradiation, the goethite particles did not dissolve within 100 s.

However, when exposed to an electron flux density of 21 e^−^ Å^−2^ s^−1^, the relative change of some particle dimensions corresponding to the width dimensions quickly decreased by ≈20% (Figure S3a, Supporting Information). Not all dimensions decreased equally and even after 100 s of beam exposure in some cases only small differences (< 5% change) were observed at this electron density. Nevertheless, the relative change in width was always larger than in length, indicating a more pronounced dissolution of the particle widths compared to dissolution at the tips of the goethite nanocrystals. Overall, from all observed dissolution experiments we evaluated that the relative change in width was −14±6% (error estimated by the standard deviation of the sample size value) and in length −3±3% after 100 s of irradiation at an electron flux density of 21 e^−^ Å^−2^ s^−1^ (Figure 1c).

In contrast, when particles were irradiated with an electron flux density of 167 e^−^ Å^−2^ s^−1^, the relative change in width follows the relative change in length across the full time of exposure from 0 to 100 s (Figure S3b, Supporting Information). Overall, at an electron flux density of 167 e^−^ Å^−2^ s^−1^, the averaged relative change in width was −45±18% and in length −31±15% (Figure 1c).

These quantified relative changes in dissolution of the goethite nanocrystals from the LP‐TEM experiments were then compared to bulk experiments consisting of equivalent goethite particles that were exposed to acidic, reductive, and alkaline conditions.

### 2.2 Bulk Acidic, Basic, and Reductive Dissolution of Goethite Particles

**Figure 2:**
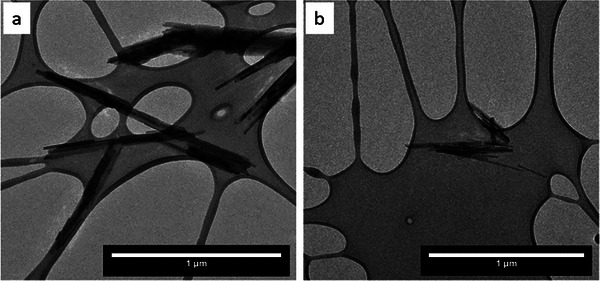
TEM micrograph of a) pristine acicular goethite particles and b) goethite particles exposed to 1 m hydroxylamine for 24 h.

Observed by conventional TEM, the pristine synthetic goethite particles also have a typical acicular crystal habit (**Figure** **2**a). After 24 h of exposure to either bulk basic or acidic pH (i.e., solutions with low and high concentration of H^+^, respectively), or to bulk reducing conditions (i.e., progressively increasing concentration of hydroxylamine NH_2_OH), the initial acicular shapes remained conserved (Figure 2b), as observed in situ, but the length and width of the particles decreased.

**Figure 3:**
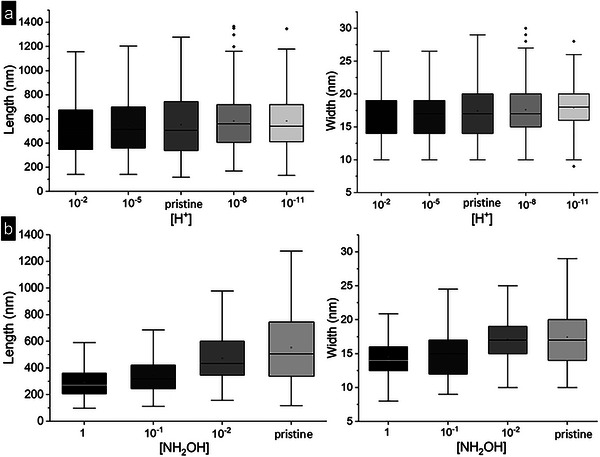
a) Box plots for the length and width of pristine non‐reacted nanoparticles, and nanoparticles exposed to pH 2, 5, 8, and 11. b) Box plots for the length and width of pristine nanoparticles, nanoparticles exposed to 1, 0.1, and 0.01 m hydroxylamine NH_2_OH. Outliers of the box plots are defined by values above the 75th Percentile + (1.5 * Interquartile Range) and under the 25th Percentile ‐ (1.5 * Interquartile Range). Lower Inner Fence = 25th Percentile ‐ (1.5 * Interquartile Range).

Histograms of the width and length dimensions enabled us to compare the dimensions of 200‐400 particles exposed to various conditions with those measured on pristine goethite particles. Figure S4 (Supporting Information) shows the size distributions obtained for pristine initial particles, as well as particles exposed to acidic (pH 2 and 5 – corresponding to 10^−2^ m H^+^ and 10^−5^ m H^+^) and basic (pH 8 and 11 – corresponding to 10^−8^ m H^+^ and 10^−11^ m H^+^) conditions for 24 h. Box plots allowed to visualize the evolution of the size distribution during acidic and basic treatment (**Figure** **3**a), and in reducing conditions (Figure 3b). In both acidic and reducing conditions, the mean and median of the width and length dimensions decreased with decreasing pH and increasing reducing conditions . The decrease in size is clearer for reducing conditions then for acidic conditions.

**Figure 4:**
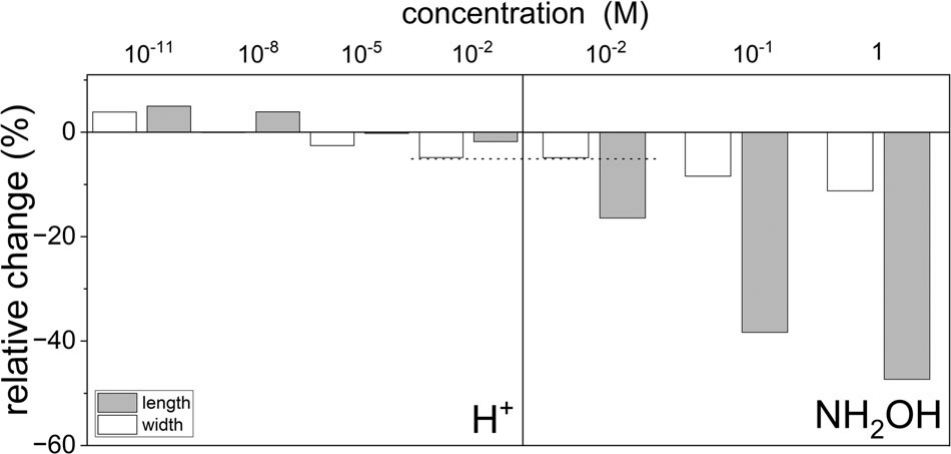
Weighted relative change in width and length of particles exposed to acidic and reductive environments. Dashed line represent the relative change in width for [H^+^]=[NH_2_OH]=0.01 m ([H^+^] equivalent to pH 2). The standard error of the mean is plotted as error bar. Note, that in most cases, this overlaps with the line thickness of the bar plot.

To account for the size distribution of the particles exposed to acidic solutions, the weighted average of the widths and lengths of the particles exposed to the respective condition (measured dimensions after treatment) was compared to the weighted average of the dimensions of pristine goethite particles. The weighted arithmetic average of a dimension *L* corresponds to:

(2) $$L\;=\frac{\sum_{i}L_{i}n_{i}}{n_{T}}\;$$

with *L*_i_ denoting the measured dimension of interest (width or length), *n*_i_ the frequency of this dimension in the population, and *n*_T_ the total number of measured dimensions. Despite the analytical limitations imposed by the broad particle size distribution (Figure 3; Figures S4 and S5, Supporting Information), the average is a reliable measure to account for the whole distribution of size trends (**Figure** **4**). The standard error of the mean was used to estimate the standard deviation of sampling distribution in each sample. These errors were used to calculate the errors on relative changes of particle dimensions upon treatment. However, as they varied between 0.02 and 0.03, they are hardly visible in Figure 4. Nevertheless, the relative changes in size still provide valuable insights into the chemical dissolution during LP‐TEM. Therefore, we focused on the general morphological trends using the average particle dimensions. A negative relative change describes a decrease in widths and lengths as compared to the initial pristine particles. Under acidic conditions, the relative change was slightly negative: particles exposed to the most acidic solution (pH 2; 10^−2^ m H^+^) had a relative change in width of maximum −5% and maximum −2% in length. With increasing pH (e.g., pH 5; 10^−5^ m H^+^), the relative change was smaller (maximum −2% in width, and close to 0% in length. Consequently, in both cases, acidic dissolution involved a higher extent of relative change in width than in length.

On the contrary, the relative changes evaluated from the experiments in alkaline conditions were positive or null: 0% and +4% in width and length for particles exposed to pH 8 (10^−8^ m H^+^), and +4% and +5%, respectively, in width and length for particles exposed to pH 11 (10^−11^ m H^+^). The positive values indicate that the resulting particles became larger than the as synthesized ones.

As the total amount of iron in our system remained constant, this suggests a dissolution reprecipitation mechanism in alkaline conditions. Deprotonation of the surface ‐OH^−^ may trigger the detachment of a surface anionic group into the solution.^[^ ^45^ ^]^ On the other hand, the precipitation of iron oxide by condensation of cations in aqueous solution occurs from pH > 1 for ferric and pH > 6 for ferrous complexes^[^ ^51^ ^]^ as predicted by thermodynamics from the Pourbaix potential‐pH diagram (Figure S2, Supporting Information). Therefore, the increase in length and width dimensions under basic conditions may be due to the dissolution of smaller goethite particles and the subsequent reprecipitation of any dissolved iron on the surface of larger goethite particles (cf. Ostwald ripening). The frequency plot of the particle size distribution exposed to pH 11 (Figure S4, Supporting Information) confirms this observation. On average the particle dimensions at pH 8 and 11 were larger after exposure to alkaline conditions.

On the other hand, the size histograms of goethite particles exposed to reducing conditions (0.01, 0.1, and 1 m hydroxylamine NH_2_OH; Figures 3b and 4; Figure S5, Supporting Information) revealed that both the length and width dimensions were significantly changed with increasing concentration of the reducing agent hydroxylamine compared to the pristine initial material. In the presence of 0.01 m hydroxylamine, the relative change was −5% in width and −16% in length and this progressively increased reaching a relative change of −11% in width and −47% in length when exposed to 1 m NH_2_OH. Therefore, for goethite particles exposed 24 h to reducing conditions, the dissolution is far more pronounced (maximum 47% reduction compared to the pristine non‐reacted goethite dimensions) than for an exposure to acidic conditions (maximum 5% of the initial dimensions). In addition, in contrast to acidic and basic exposures, the reductive dissolution lead to a smaller relative change in width than in length of the goethite particles.

The results obtained from the basic, acidic, and reductive dissolution experiments show that, apart from the reprecipitation process observed for basic conditions, two distinct dissolution processes can be assigned to the exposure to acidic or reductive conditions influencing the morphological evolution though time. Indeed, for a similar concentration of H^+^ or NH_2_OH (10^−2^ m), the relative change in width was similar: 5%, however, in presence of high H^+^ concentration (pH 2) the relative change in length was −2%, whereas under reducing conditions, the extent of relative change in length was larger: −16%. We have exploited these relative changes in length and width under acidic and reducing conditions to investigate the chemistry of the surrounding aqueous solution.

### 2.3 In Situ Enhanced Acidic Dissolution of Goethite Particles

The evaluated changes in goethite nanocrystal dimensions in the in situ experiment were compared to the results from our acidic, basic, and reducing bulk experiments to probe the effects of radiation chemistry. In both sets of experiments, we determined dissolution rates and compared these with bulk literature values.

First, for low electron dose rates in the liquid cell (i.e., 21 e^−^ Å^−2^ s^−1^ and Figure 1c), the relative change in particle lengths (‐3±3%,) was lower than in widths (‐14±6%), therefore the trend was the same as in the ex situ bulk acidic experiments (Figure 4). Nevertheless, the relative changes in the in situ experiments at low electron flux densities were twice as high as the values obtained for a 24 h exposition to acidic solution at pH 5. Thus, although the suggests dissolution via acidification of the surrounding medium, at these electron flux densities other factors may also influence or at least enhance acidic dissolution.

On the other hand, at high electron flux densities (i.e., 167 e^−^ Å^−2^ s^−1^), the extent of the relative change in lengths (−31±15%) and widths (−45±18%) were larger compared to the low electron density, thus more clearly suggesting dissolution of the goethite particles. Yet again, the extent of relative change was ten times larger than the one observed in the equivalent bulk experiment at an acidic pH of 5 or lower.

According to the loss of volume in goethite needles during the 100 s of electron beam‐induced dissolution at high electron flux densities, 2.8 10^−18^ mol of iron were released into the surrounding medium. Thus, dissolution occurred at a rate of 1.64 10^−1^ g Fe h^−1^ m^−2^ (further details for this calculation in Text and Figure S6, Supporting Information). Comparison with dissolution rates derived from our bulk experiments and to values previously reported in literature (Table S3, Supporting Information) revealed that the dissolution rates obtained in our LP‐TEM experiments were 3 to 5 orders of magnitude larger than those found for non‐irradiated dissolution in the present study (strongest reductive conditions (1 m NH_2_OH): 3.64 10^−4^ g Fe h^−1^ m^−2^ and the most acidic conditions (pH 2): 1.18 10^−5^ g Fe h^−1^ m^−2^). Reported values for goethite dissolution were between 1.00 10^−6^ g Fe h^−1^ m^−2^ in 0.5 m HCl,^[^ ^46^, ^52^, ^53^ ^]^ to 1.03 10^−4^ g Fe h^−1^ m^−2^ under reducing conditions (under 30 mm sodium dithionite addition).^[^ ^54^ ^]^ This up to 4‐5 orders of magnitude large discrepancy between the rates derived from our in situ LP‐TEM experiments and our and the literature bulk dissolution rates is a consequence of the effect of the beam induced radiation chemical changes during LP‐TEM. Such differences between liquid cell and bulk experiment dissolution rates have been previously observed in other systems as well. For example, a dissolution rate of about five orders of magnitude higher was evidenced during cerium dioxide nanoparticle dissolution in LP‐TEM as compared to non‐beam‐induced ex situ experiments.^[^ ^55^ ^]^

### 2.4 Model Comparison

The experimental results were then compared to simulation of the radiolytic products formed under electron irradiation in the liquid cell. Previous studies on the irradiation of pure water with an incident electron beam indicate a decrease in water pH down to 3 within seconds.^[^ ^17^ ^]^ However, radiolysis of aqueous phases with electrons does not only produce H^+^, but also OH^−^:^[^ ^56^ ^]^

(3) $$H_{2}O\;\overset{\begin{array}{c} \mathrm{ionizing}\\ \mathrm{radiation} \end{array}}{\to }\begin{array}{c} e_{h}^{-},{\;\mathrm{HO}}^{\cdot },{\;H}^{\cdot },{\;\mathrm{HO}}_{2}^{\cdot },\\ H^{+},{\;\mathrm{OH}}^{-},{\;H}_{2}O_{2},{\;H}_{2} \end{array}\;$$

By neglecting this concomitant increase in OH^−^ concentration during electron irradiation, the actual conditions during LP‐TEM are often misinterpreted by simply using the standard proxy of pH = lg([H^+^]). To overcome this problem, we recently suggested a novel approach to evaluate the acidity or basicity of the irradiated medium. As such, the radiolytic acidity π* alongside with the radiolytic ion product *K*_w_* was introduced. Those relate to both, [H^+^] and [OH^−^] under exposure to ionizing radiation:^[^ ^57^ ^]^

(4) $$\pi^{*}=\;\mathrm{lg}\left(\frac{\left[H^{+}\right]}{\left[{\mathrm{OH}}^{-}\right]}\right)\;$$

(5) $$K_{W}^{*}={\left(\left[H^{+}]\cdot [{\mathrm{OH}}^{-}\right]\right)}_{\mathrm{irradiated}\;}\;$$

Consequently, a negative π* describes a basic solution, and a positive π* describes an acidic solution, while *K*_w_* is a measure of the magnitude of the acidic / basic environment. While being already applied recently,^[^ ^57^, ^58^ ^]^ this concept has not been validated against experimental data yet.

Although simulations of radiation chemistry in an isotropic voxel approximation invariably have to neglect phase boundaries, diffusion and spur effect from the scanning beam which may cause transients^[^ ^19^ ^]^ and delay steady state formation.^[^ ^17^, ^30^ ^]^ Nonetheless, such simulations provide valuable insights into the radiation chemistry during LP‐TEM studies and are therefore suited to gauge the evolution of the water chemistry under irradiation if a suitable reaction network is defined.

**Figure 5:**
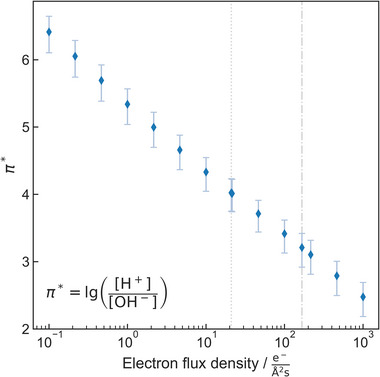
Radiolytic acidity π* as a function of electron flux density assuming a Fe^3+^ release during dissolution. The vertical lines denote the investigated in situ conditions. The error bars derive from additional simulations based on the upper and lower value obtained from the uncertainty in released iron concentration of (4.8±1.4 mM). The inset formula corresponds to Equation (4).

To test this for our system, iron was incorporated into a reaction network of pure water that we have developed and validated recently.^[^ ^19^, ^57^ ^]^ The concentration of iron species was derived from the total amount of iron released in the surrounding medium in our in situ experiments. Using the irradiated volume of the liquid cell, the resulting concentration in the surrounding medium amounts to 4.8±1.4 mM. As elucidated above, goethite could either dissolve via a reductive or an acidic pathway. The former and the later pathway would result in the release of Fe^2+^ or Fe^3+^, respectively, in the surrounding solution. Both situations were modeled (**Figure** **5**; Figures S7 and S8, Supporting Information) and compared to the experimental findings discussed above.

#### 2.4.1. Case I: Fe is exposed to Radiation Chemistry as Fe^2+^

At both electron flux densities considered, the radiolytic acidity of the system in the presence of dissolved ferrous iron is negative (Figure S7, Supporting Information). This would suggest a basic environment. However, this contrasts with experimental observation. As no precipitate was observed inside or outside the viewing window, neither in situ, nor post situ, it is unlikely that the established steady state corresponds to a basic environment. Thus, it is unlikely that Fe^2+^ is the primary oxidation state in the context of irradiation‐mediated goethite dissolution.

#### 2.4.2. Case II: Fe is exposed to Radiation Chemistry as Fe^3+^

At low electron flux density, π* would correspond to ≈4 (Figure 5), denoting an overall acidic solution. This result is in good agreement with the morphological observation features (i.e., the dissolution of particle width is higher than the dissolution in length). Hence it confirms that acidic dissolution occurs at low electron dose rate.

At higher electron flux densities, the experimental findings do not unambiguously suggest an acidic dissolution pathway. This could be explained with either case, as at the related dose rate π* is driven toward neutral conditions (lower π* in Figure 5). Simultaneously, the (excess) steady state concentrations of strong reductants (i.e., H radical and e_h_^−^) rise notably (Figure S8, Supporting Information). As shown in Table S2 (Supporting Information) (reactions 92 and 94), these species dominate Fe^3+^ reduction, which is driving the release of iron from the crystal structure during reductive dissolution. This could trigger a superposition of the two possible dissolution pathways and, thus, explain both, the observed unambiguity, and the observed increase in dissolution rate during LP‐TEM.

This hypothesis is supported by the presence of ferrous iron in the simulation set (Figure S8, Supporting Information). Fe^3+^ is continuously reduced to ferrous Fe^2+^ during beam irradiation in the aqueous solution. Subsequently, the simultaneous presence of oxidative species (i.e., the OH radical) triggers a dynamic redox equilibrium in which Fe^2+^ appears to be complexed to [FeOH]^2+^. Therefore, all steady state concentrations only account for the remaining net concentrations after reaching this dynamic equilibrium and do not fully capture the reactivity of the solution before reaching the steady state.

Moreover, this emphasizes once more the need for tailored and combined radiation chemistry experiment and simulations that reflect the solution chemistry inside a cell during LP‐TEM experiments. This should include also the interaction with solutes instead of relying solely on modeling the radiation chemistry of the pristine solvent only.

## 3 Conclusion

The present study successfully demonstrates that probing the changes in aqueous media chemistry during LP‐TEM experiments using goethite as a marker system is feasible. By comparing the morphological evolution of the goethite particle in situ to the evolution obtained under various bulk chemical environments (acidic, basic, and reductive) a holistic understanding of the chemical dissolution conditions under beam irradiation inside a LP‐TEM cell was achieved. Our observations were also cross‐correlated with kinetic simulations of the radiolytic species in the irradiated surrounding aqueous solution.

Both experiments and simulations confer that the absence of solid forming on the particle or in the surrounding medium make an alkaline goethite dissolution during beam exposure inside of an LP‐TEM improbable. All morphological evolutions evaluated suggest an acidic dissolution of the particle at lower electron fluxes, which was also confirmed by the simulations. On the other hand, the morphological evolution during the in situ and bulk dissolution experiments in the presence of reducing agents combined with the simulation indicated that the surrounding solution was less acidic and suggested an increased contribution of the reductant molecules in the aqueous medium.

Furthermore, the dissolution rates were notably enhanced in situ as compared to the bulk chemical experiments, confirming that an additional contribution from radiolytic reactive species emerging with an increased electron flux density has to be taken into consideration and, showcasing the requirement of more suitable radiation chemical experiments and models. Hence, the results open up new perspectives in the design and implementation of other in situ nanoparticulate indicators dissolving or precipitating at different acidity or redox conditions to further investigate the solvent chemistry during LP‐TEM imaging. Finally, the results also provide a significant technical improvement toward the study of dissolution processes involved industrial and natural settings.

## 4 Experimental Section

### Goethite Synthesis

Goethite was synthesized using a modified method for aqueous ageing of ferrihydrite which was suggested by Schwertmann et al.^[^ ^59^ ^]^ and Chen.^[^ ^60^ ^]^ Sodium hydroxide (NaOH) solution (5 m, 120 ml) was added dropwise to an iron (III) nitrate solution (1 m, 200 ml) while stirring in a 500 ml polypropylene bottle. The resulting ferrihydrite suspension was basified by dropwise addition of sodium hydroxide (5 m) until pH 12 was reached. The bottle was then closed and heated in a furnace for three days. The initial reaction temperature was 50 °C, which was increased to 60 °C after the first day. The product was recovered by centrifugation (5 min, 7690 g (1 g = 9.81 m s^−2^)) and subsequently washed by resuspension in water followed by another centrifugation, repeated four times. After drying (50 °C, 3 days) and grinding in an agate mortar the final product was obtained as a yellow powder (17.23 g, 97% yield).

### Bulk Acidic, Basic, and Reductive Goethite Dissolution

For acidic dissolution, goethite particles (0.4 g L^−1^) were exposed to ultrapure water at pH 2 and 5 (adjusted using 1 m HCl and 1 m NaOH) for 24 h. For basic dissolution, similar experiments at pH 8 and 11 were conducted, but the reaction proceeded under argon flow to prevent acidification from atmospheric CO_2_ dissolution. At the end of each experiment, the solids were separated from the solution by vacuum filtration (Whatman, polycarbonate, 0.2 µm), and dried in a desiccator for 24 h. Particles were then characterized using FIB‐SEM and conventional TEM.

For reductive dissolution, goethite particles (0.4 g L^−1^) were exposed to 1, 0.1, and 0.01 m hydroxylamine (NH_2_OH) in ultrapure water for 24 h. The pH was adjusted to 5 using 1 m HCl and 1 m NaOH. The solids were extracted from the solution by vacuum filtration (Whatman, polycarbonate, 0.2 µm), and placed in the desiccator for further 24 h prior to TEM characterization.

### TEM Analysis

Goethite particles were analyzed using a Thermo Fisher Scientific (former FEI) Tecnai G2 F20 X‐Twin transmission electron microscope equipped with a field‐emission gun. TEM samples were prepared by suspending a few mg of powdered sample in 1 ml of acetone, ultrasonicating for 30 s, then drop‐casting onto a holey carbon Cu TEM grid. Micrographs were acquired at an acceleration voltage of 200 kV as energy‐filtered images on a Gatan GIF Tridiem detector. The width and length dimensions of 200‐400 goethite particles from each experiment were quantified using ImageJ (version 1.52a).^[^ ^61^ ^]^ The particle dimensions were measured on isolated particles (and not aggregates) to ensure that the TEM images correspond to a reliable 2D projection of each particle on the TEM grid.

### Liquid‐Phase TEM Experiment

The LP‐TEM experiments were carried out in a dedicated specimen holder (Protochips Inc., Poseidon 300) using microchips for confining the liquid specimen between two SiN*_x_* membranes with an electron transparent window area of 550 × 50 µm^2^ in cross configuration and separated by 150 nm gold spacers. Bulging of these thin membranes creates a range of liquid thicknesses within the same cell, impacting the spatial resolution. For the in situ experiments, goethite particles were imaged in the corners of the resulting electron transparent viewing area to minimize the liquid thickness surrounding the particles and optimize spatial resolution.

Prior to the liquid cell mounting, goethite particles were suspended in acetone and drop casted onto the silicon chips to immobilize the solid specimen on the electron transparent window. After evaporation of the acetone, 2 µL of water at pH 5.5 were deposited prior to encapsulation. Data acquisition was performed at an acceleration voltage of 200 kV in STEM (scanning transmission electron microscopy) mode by using a high‐angle annular dark‐field (HAADF) detector, a 30 µm condenser aperture and a spot size to maintain a constant beam current of 3.19 nA (measured in vacuum before introduction of the liquid cell). All micrographs were recorded in a 512 by 512 pixel format with a pixel dwell time of 10 µs. The two electron fluxes 21 and 167 e^−^ Å^−2^ s^−1^ were calculated according to the following equation:^[^ ^62^ ^]^

(6) $$d\;=\frac{I}{e\;S}$$

where *d* is the continuous electron flux received per second by the sample, e^−^ Å^−2^ s^−1^, *I* is the beam current in C s^−1^, *e* the elementary charge, in C e^−^ and *S* the irradiated surface in Å. Therefore, at a fixed intensity, the electron flux depends on the irradiated surface, and therefore only on the magnification of the electron microscopy image.

Suitable serial image acquisition conditions were achieved by focusing away from the area of interest, followed by blanking of the beam and translation to the area of interest. This procedure guaranteed that the area of interest was not exposed to electrons prior to scanning the first frame. A background intensity gradient visible in the images was likely related to the variation in fluid thickness. The presence of water in the liquid cell was monitored via the oxygen signal in EDX spectra and additionally confirmed by the observation of a water droplet on the SiN_x_ membrane, when the liquid cell was disassembled after the in situ analysis. All LP‐TEM experiments were performed in extremely small sample volumes (2 µl) and low nanoparticle concentrations. In addition, the probability of finding an area of interest was further reduced as the clearest images (best spatial resolution) are usually obtained in the corners of the crossed viewing windows of the liquid cell. In this study, in situ dimensions changes of four goethite particles from two independent, but equivalent experiments are reported. Furthermore, the size measurements were only performed on isolated goethite particles to prevent any misorientation of particles that were aggregated on the TEM grids.

### Kinetic Modeling

Modeling of the changes in solution chemistry was performed using AuRaCh, a Python‐based tool for automated simulation of radiation chemistry.^[^ ^9^ ^]^ The formation of primary species was accounted for by generation values listed in Table S1 (Supporting Information). To account for the interplay between the irradiated aqueous solution products and the Fe‐based species, the reaction network of pure water comprising 83 coupled reactions^[^ ^19^ ^]^ was appended by thirteen reactions denoting the interplay with Fe^2+^, Fe^3+^, and [FeOH]^2+^ species^[^ ^63^, ^64^, ^65^, ^66^, ^67^, ^68^, ^69^ ^]^(see Table S2, Supporting Information). The AuRaCh‐compatible reaction set is available at the AuRaCh GitHub repository (https://github.com/BirkFritsch/Radiolysis‐simulations).

In all simulations, an isotropic voxel was assumed, suggesting a rapid steady state formation (Figure S1, Supporting Information). This approach allowed for a direct comparison of the steady‐state concentrations obtained as a function of the dose rate *ψ*. To convert the electron flux density ϕ to *ψ*, the following formula was utilized:^[^ ^70^ ^]^

(7) $$\psi \;=\left(1+\frac{z_{l}}{\lambda_{\mathrm{IMFP}}}\right)\;S\frac{\phi }{e\;}$$

assuming a liquid thickness *z*_l_ of 2.2 µm, as obtained via STEM defocus measurement in a comparable set up,^[^ ^70^ ^]^ an inelastic mean free path λ_IMFP_ of ≈320 nm,^[^ ^71^ ^]^ and a stopping power *S* of 2.798 MeV cm^2^ g^−1^.^[^ ^72^ ^]^ *e* denotes the elementary charge. To relate to our experiments, the pH prior to irradiation was set to 5.5. Aeration was accounted for by an initial O_2_ concentration of 255 µM.

### Thermodynamic Modeling

Thermodynamic calculations, including Pourbaix diagrams were performed with Geochemist's Workbench Community Edition 15.0 software, implementing the thermo.tdat thermodynamic database for aqueous mineral and gas reactions.^[^ ^73^ ^]^

## Conflict of Interest

The authors declare no conflict of interest.

## Author Contributions

T.C. performed all the TEM experiments and bulk chemical dissolution studies and calculated the Pourbaix diagrams. B.F. performed the radiation chemistry modeling. R.B. synthesized the goethite particles. T.C., B.F., and A.H. wrote the initial manuscript, and all authors provided guidance and edited the final version of the manuscript.

## Supporting information

Supporting Information

Supplemental Video 1

Supplemental Video 2

## Data Availability

The data that support the findings of this study are openly available at https://www.github.com/BirkFritsch/Radiolysis‐simulations, reference number 1. These data were derived from the following resources available in the public domain: [Resource 1], https://www.github.com/BirkFritsch/Radiolysis‐simulations; [Resource 2], https://www.github.com/BirkFritsch/Radiolysis‐simulations.
