# Supplementary material for: Goethite Mineral Dissolution to Probe the Chemistry of Radiolytic Water in Liquid‐Phase Transmission Electron Microscopy
Source: Adv Sci (Weinh). 2023 Jul 13;10(25):2301904. doi: 10.1002/advs.202301904 (PMC10477898; doi:10.1002/advs.202301904)
Supplement: Supplementary file 1 — Supporting Information [file ADVS-10-2301904-s001.pdf]

ADVANCED SCIENCE
Open Access

Supporting Information

for *Adv. Sci.*, DOI 10.1002/advs.202301904

Goethite Mineral Dissolution to Probe the Chemistry of Radiolytic Water in Liquid-Phase Transmission Electron Microscopy

Thaïs Couasnon, Birk Fritsch, Michael P. M. Jank, Roberts Blukis, Andreas Hutzler* and Liane G. Benning*

Electronic Supplementary Information for the manuscript: Goethite Mineral Dissolution to Probe the Chemistry of Radiolytic Water in Liquid-Phase Transmission Electron Microscopy

Thaïs Couasnon, Birk Fritsch, Michael P. M. Jank, Roberts Blukis, Andreas Hutzler and Liane G. Benning

Tables S1, S2 and Figure S1 Isotropic voxel simulations

Table S1*:* Generation values used for radiolysis simulations*[1]*.

primary species	Generation value [Molecules/100 eV]
e_h^-	3.47
H^+	4.42
OH^-	0.95
H_2O_2	0.47
H	1.00
OH	3.63
HO_2	0.08
H_2	0.17
H_2O	-5.68

Table S2: Kinetic model to describe radiolysis of aqueous solutions containing Fe^{3+} with *k* as rate constant in units of $mol^{-n+1}\ dm^{3(n-1)}\ s^{-1}$. *n* denotes the reaction order. The first 83 reactions stem from ref.[2]. Involved atomic, ionic and radical species presented in the reaction set corresponds to the following species: H+: Hydrogen ion (H^+, CAS 12408-02-5), H: Hydrogen radical($H^\cdot$, CAS 12385-13-6), H_2O_2: Hydrogen peroxide (H_2O_2, CAS 7722-84-1), HO_2: Hydroperoxyl ($HO_2^\cdot$, CAS 3170-83-0), HO_2^-: Hydroperoxide ion (HO_2^-, CAS 14691-59-9), H_2O: Water (H_2O, CAS 7732-18-5), OH: Hydroxyl radical ($OH^\cdot$, CAS 3352-57-6), OH^-: Hydroxide anion (OH^-, CAS 14280-30-9), O^-: Oxide radical ion ($O^{\cdot-}$, CAS 14337-01-0), O_2: Molecular oxygen (O_2, CAS 7782-44-7), O_2^-: Superoxide ($O_2^{\cdot-}$, CAS 11062-77-4), O_3: Ozone (O_3, CAS 10028-15-6), O_3^-: Ozonide ion ($O_3^{\cdot-}$, CAS 12596-80-4), $[FeOH]^{2+}$: Hydroxoiron(III) ion ($FeOH^{2+}$, CAS 12299-69-3), Fe^{2+}: Iron(II) ion (Fe^{2+}, CAS 15438-31-0), Fe^{3+}: Iron(III) ion (Fe^{3+}, CAS 20074-52-6).

	Reaction			*k* [$mol^{-n+1}\ dm^{3(n-1)}\ s^{-1}$]	Source
1	H_2O	→	$H^+ + OH^-$	$2.599 \cdot 10^{-5}$	[2]
2	$H^+ + OH^-$	→	H_2O	$1.43 \cdot 10^{11}$	[2]
3	H_2O_2	→	$H^+ + HO_2^-$	$1.119 \cdot 10^{-1}$	[2]
4	$H^+ + HO_2^-$	→	H_2O_2	$5 \cdot 10^{10}$	[2]

5	$H_2O_2 + OH^-$	→	$HO_2^- + H_2O$	$1.3 \cdot 10^{10}$	[2]
6	$HO_2^- + H_2O$	→	$H_2O_2 + OH^-$	$5.82 \cdot 10^{7}$	[2]
7	$e_h^- + H_2O$	→	$H + OH^-$	$1.9 \cdot 10^{1}$	[2]
8	$H + OH^-$	→	$e_h^- + H_2O$	$2.2 \cdot 10^{7}$	[2]
9	H	→	$e_h^- + H^+$	$3.9 \cdot 10^{0}$	[2]
10	$e_h^- + H^+$	→	H	$2.3 \cdot 10^{10}$	[2]
11	$OH + OH^-$	→	$O^- + H_2O$	$1.3 \cdot 10^{10}$	[2]
12	$O^- + H_2O$	→	$OH + OH^-$	$1 \cdot 10^{8}$	[2]
13	OH	→	$O^- + H^+$	$1.259 \cdot 10^{-1}$	[2]
14	$O^- + H^+$	→	OH	$1 \cdot 10^{11}$	[2]
15	HO_2	→	$O_2^- + H^+$	$1.346 \cdot 10^{6}$	[2]
16	$O_2^- + H^+$	→	HO_2	$5 \cdot 10^{10}$	[2]
17	$HO_2 + OH^-$	→	$O_2^- + H_2O$	$5 \cdot 10^{10}$	[2]
18	$O_2^- + H_2O$	→	$HO_2 + OH^-$	$1.862 \cdot 10^{1}$	[2]
19	$e_h^- + OH$	→	OH^-	$3 \cdot 10^{10}$	[2]
20	$e_h^- + H_2O_2$	→	$OH + OH^-$	$1.1 \cdot 10^{10}$	[2]
21	$e_h^- + O_2^- + H_2O$	→	$HO_2^- + OH^-$	$1.3 \cdot 10^{10}$	[2]
22	$e_h^- + HO_2$	→	HO_2^-	$2 \cdot 10^{10}$	[2]
23	$e_h^- + O_2$	→	O_2^-	$1.9 \cdot 10^{10}$	[2]
24	$2\ e_h^- + 2\ H_2O$	→	$H_2 + 2\ OH^-$	$5.5 \cdot 10^{9}$	[2]
25	$e_h^- + H + H_2O$	→	$H_2 + OH^-$	$2.5 \cdot 10^{10}$	[2]
26	$e_h^- + HO_2^-$	→	$O^- + OH^-$	$3.5 \cdot 10^{9}$	[2]
27	$e_h^- + O^- + H_2O$	→	$OH^- + OH^-$	$2.2 \cdot 10^{10}$	[2]
28	$e_h^- + O_3^- + H_2O$	→	$O_2 + OH^- + OH^-$	$1.6 \cdot 10^{10}$	[2]
29	$e_h^- + O_3$	→	O_3^-	$3.6 \cdot 10^{10}$	[2]
30	$H + H_2O$	→	$H_2 + OH$	$1.1 \cdot 10^{1}$	[2]
31	$H + O^-$	→	OH^-	$1 \cdot 10^{10}$	[2]
32	$H + HO_2^-$	→	$OH + OH^-$	$9 \cdot 10^{7}$	[2]
33	$H + O_3^-$	→	$OH^- + O_2$	$1 \cdot 10^{10}$	[2]
34	$2\ H$	→	H_2	$7.8 \cdot 10^{9}$	[2]
35	$H + OH$	→	H_2O	$7 \cdot 10^{9}$	[2]
36	$H + H_2O_2$	→	$OH + H_2O$	$9 \cdot 10^{7}$	[2]
37	$H + O_2$	→	HO_2	$2.1 \cdot 10^{10}$	[2]
38	$H + HO_2$	→	H_2O_2	$1.8 \cdot 10^{10}$	[2]
39	$H + O_2^-$	→	HO_2^-	$1.8 \cdot 10^{10}$	[2]
40	$H + O_3$	→	HO_3	$3.8 \cdot 10^{10}$	[2]
41	$2\ OH$	→	H_2O_2	$3.6 \cdot 10^{9}$	[2]
42	$OH + HO_2$	→	$H_2O + O_2$	$6 \cdot 10^{9}$	[2]

43	$OH + O_2^-$	→	$OH^- + O_2$	$8.2 \cdot 10^9$	[2]
44	$OH + H_2$	→	$H + H_2O$	$4.3 \cdot 10^7$	[2]
45	$OH + H_2O_2$	→	$HO_2 + H_2O$	$2.7 \cdot 10^7$	[2]
46	$OH + O^-$	→	HO_2^-	$2.5 \cdot 10^{10}$	[2]
47	$OH + HO_2^-$	→	$HO_2 + OH^-$	$7.5 \cdot 10^9$	[2]
48	$OH + O_3^-$	→	$O_3 + OH^-$	$2.6 \cdot 10^9$	[2]
49	$OH + O_3^-$	→	$2\ O_2^- + H^+$	$6 \cdot 10^9$	[2]
50	$OH + O_3$	→	$HO_2 + O_2$	$1.1 \cdot 10^8$	[2]
51	$HO_2 + O_2^-$	→	$HO_2^- + O_2$	$8 \cdot 10^7$	[2]
52	$HO_2 + HO_2$	→	$H_2O_2 + O_2$	$7 \cdot 10^5$	[2]
53	$HO_2 + O^-$	→	$O_2 + OH^-$	$6 \cdot 10^9$	[2]
54	$HO_2 + H_2O_2$	→	$OH + O_2 + H_2O$	$5 \cdot 10^{-1}$	[2]
55	$HO_2 + HO_2^-$	→	$OH + O_2 + OH^-$	$5 \cdot 10^{-1}$	[2]
56	$HO_2 + O_3^-$	→	$O_2 + O_2 + OH^-$	$6 \cdot 10^9$	[2]
57	$HO_2 + O_3$	→	$HO_3 + O_2$	$5 \cdot 10^8$	[2]
58	$2\ O_2^- + 2\ H_2O$	→	$H_2O_2 + O_2 + 2\ OH^-$	$1 \cdot 10^2$	[2]
59	$O_2^- + O^- + H_2O$	→	$O_2 + 2\ OH^-$	$6 \cdot 10^8$	[2]
60	$O_2^- + H_2O_2$	→	$OH + O_2 + OH^-$	$1.3 \cdot 10^{-1}$	[2]
61	$O_2^- + HO_2^-$	→	$O^- + O_2 + OH^-$	$1.3 \cdot 10^{-1}$	[2]
62	$O_2^- + O_3^- + H_2O$	→	$O_2 + O_2 + 2\ OH^-$	$1 \cdot 10^4$	[2]
63	$O_2^- + O_3$	→	$O_3^- + O_2$	$1.5 \cdot 10^9$	[2]
64	$2\ O^- + H_2O$	→	$HO_2^- + OH^-$	$1 \cdot 10^9$	[2]
65	$O^- + O_2$	→	O_3^-	$3.6 \cdot 10^9$	[2]
66	$O^- + H_2$	→	$H + OH^-$	$8 \cdot 10^7$	[2]
67	$O^- + H_2O_2$	→	$O_2^- + H_2O$	$5 \cdot 10^8$	[2]
68	$O^- + HO_2^-$	→	$O_2^- + OH^-$	$4 \cdot 10^8$	[2]
69	$O^- + O_3^-$	→	$O_2^- + O_2^-$	$7 \cdot 10^8$	[2]
70	$O^- + O_3$	→	$O_2^- + O_2$	$5 \cdot 10^9$	[2]
71	O_3^-	→	$O_2 + O^-$	$3.3 \cdot 10^3$	[2]
72	$O_3^- + H^+$	→	$O_2 + OH$	$9 \cdot 10^{10}$	[2]
73	HO_3	→	$O_2 + OH$	$1.1 \cdot 10^5$	[2]
74	H_2O_2	→	$H_2O + O$	$1 \cdot 10^{-3}$	[2]
75	$2\ O$	→	O_2	$1 \cdot 10^9$	[2]
76	O_3	→	$O_2 + O$	$3 \cdot 10^{-6}$	[2]
77	$2\ O_3^- + H_2O$	→	$OH^- + HO_2^- + 2\ O_2$	$1 \cdot 10^4$	[2]
78	$2\ HO_3$	→	$H_2O_2 + 2\ O_2$	$5 \cdot 10^9$	[2]
79	$O_3 + OH^-$	→	$HO_2^- + O_2$	$1 \cdot 10^2$	[2]
80	$O_2 + O$	→	O_3	$4 \cdot 10^9$	[2]

81	$H_2O_2 + O$	→	$OH + HO_2$	$1.6 \cdot 10^9$	[2]
82	$O + HO_2^-$	→	$OH + O_2^-$	$5.3 \cdot 10^9$	[2]
83	$O + OH^-$	→	HO_2^-	$4.2 \cdot 10^8$	[2]
84	$Fe^{2+} + H_2O_2$	→	$Fe^{3+} + OH + OH^-$	$5.7 \cdot 10^2$	[3]
85	$Fe^{2+} + OH$	→	$Fe^{3+} + OH^-$	$3.2 \cdot 10^8$	[3]
86	$Fe^{2+} + OH$	→	$[FeOH]^{2+}$	$3.2 \cdot 10^8$	[4]
87	$Fe^{2+} + H^+ + HO_2$	→	$H_2O_2 + Fe^{3+}$	$1.2 \cdot 10^6$	[5]
88	$Fe^{2+} + O_2^- + 2\ H^+$	→	$Fe^{3+} + H_2O_2$	$1 \cdot 10^7$	[5]
89	$Fe^{2+} + O_3$	→	$Fe^{3+} + O^- + O_2$	$8.2 \cdot 10^5$	[6]
90	$Fe^{2+} + O^- + H^+$	→	$Fe^{3+} + OH^-$	$3.8 \cdot 10^9$	[7]
91	$Fe^{3+} + HO_2$	→	$Fe^{2+} + O_2 + H^+$	$3.1 \cdot 10^7$	[3]
92	$Fe^{3+} + e_h^-$	→	Fe^{2+}	$6 \cdot 10^{10}$	[8]
93	$Fe^{3+} + H_2O_2$	→	$Fe^{2+} + O_2^- + H^+$	$2.6 \cdot 10^{-3}$	[3]
94	$Fe^{3+} + H$	→	$Fe^{2+} + H^+$	$1.2 \cdot 10^9$	[7]
95	$[FeOH]^{2+} + O_2^- + H^+$	→	$Fe^{2+} + O_2 + H_2O$	$1.5 \cdot 10^8$	[5]
96	$[FeOH]^{2+} + H$	→	$Fe^{2+} + H_2O$	$1.2 \cdot 10^9$	[9]

Isotropic voxel simulations naturally neglect phase boundaries, diffusion and spur effect from the scanning beam which can cause transients[2] and delay steady state formation[1,10]. Nonetheless, such simulations provide valuable insights into the radiation chemistry during liquid-phase electron microscopy and are therefore suited to gauge the evolution of the water chemistry under irradiation. Exemplary steady state formations are shown in Figure S1.

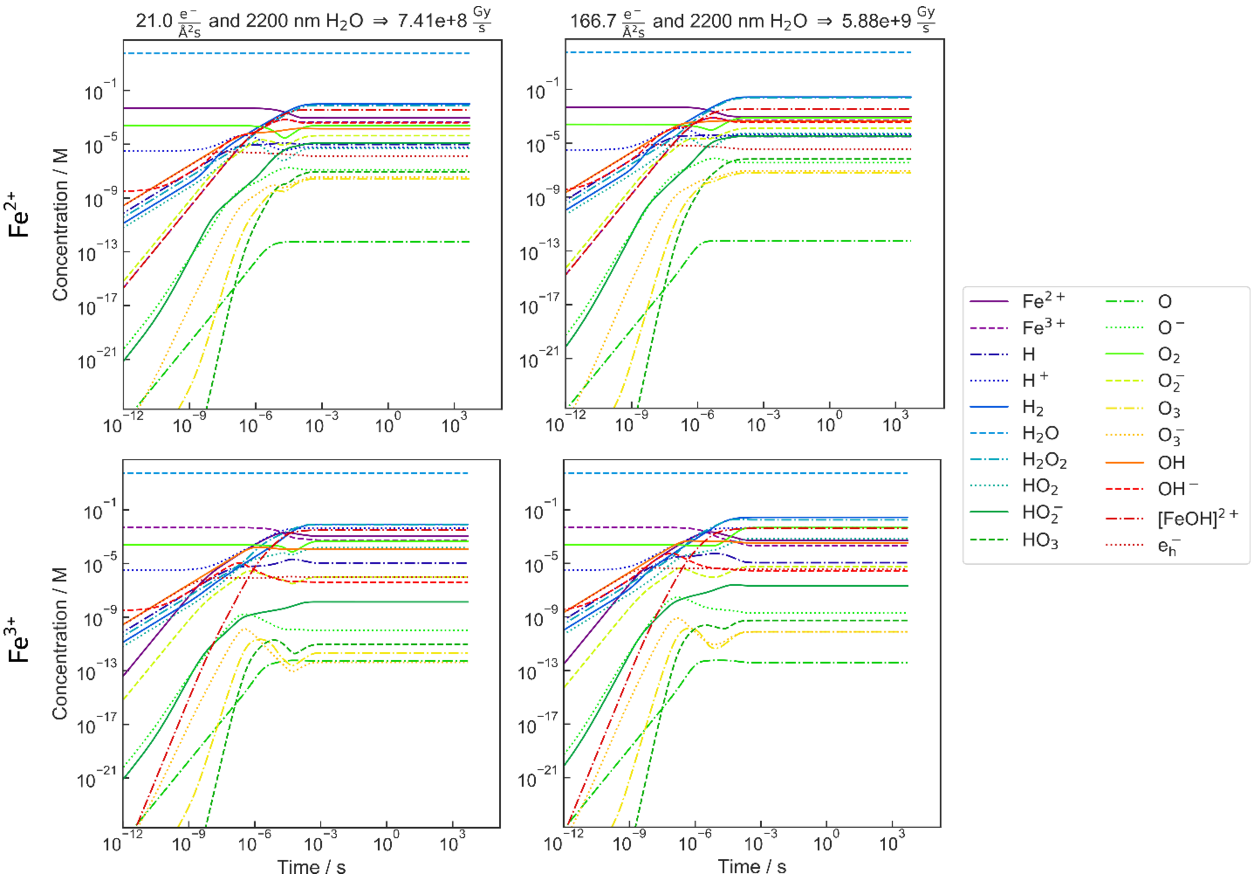

Figure S1: Temporal evolution of the species concentrations at the electron flux densities used during LP-TEM in this study.

Figure S2 Pourbaix diagram

Eh/pH Pourbaix diagram was calculated using the software core Geochemical workbench[11]. The plots in Figure S2 reveal the thermodynamically stable phases at 25 °C, under atmospheric conditions and in the presence of 4.8 mM of dissolved Fe^{3+}. The data shows that hematite (Figure S2a) should be the stable phase under our standard conditions. However, in our study we focused on goethite and at our specific LP-TEM settings (imaging parameters, electron flux, liquid cell thickness) we only observed the dissolution of goethite particle, and did not expect any transformation of the goethite to the hematite. To show this we excluded hematite as a stable phase from our Pourbaix diagram shows (Figure S2b) that indeed goethite is the predominant phase over a large range of conditions and only at pH lower than 7, the predominant species can be aqueous: either Fe^{2+} or Fe^{3+} depending on solution potential. However, it must be emphasized that one must be careful in transferring and applying such pH-dependent thermodynamic representations to conditions present in LP-TEM.

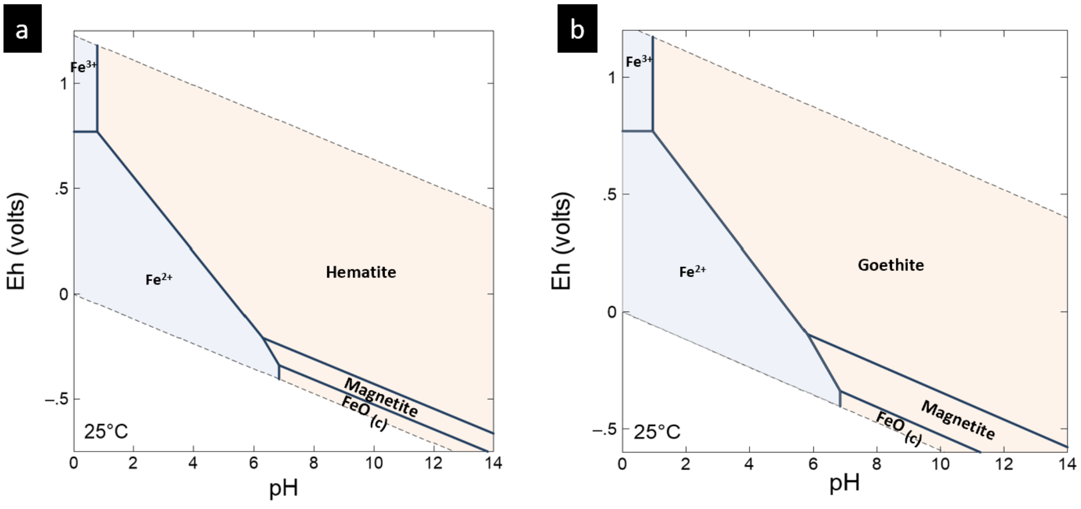

Figure S2: Pourbaix Diagram of iron at room temperature using Geochemical workbench including (a), or excluding (b) hematite. Eh refers to the oxidation-reduction potential.

Figure S3 Relative change in mineral width and length *in situ*

The dimensions were measured on 26 width and length from 4 different particles in 2 distinct independent experiments. Red and black marks correspond to length and width dimensions, respectively.

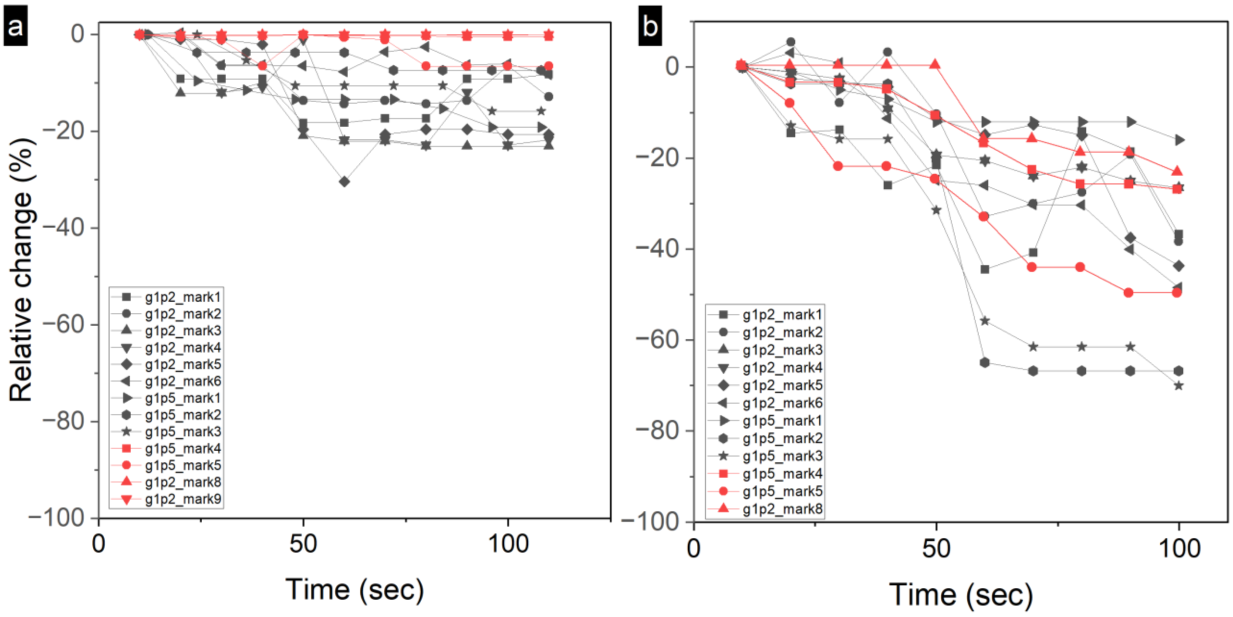

Figure S3: relative change in length and width dimension of particles observed in situ with an electron flux density of (a) 21 e^{-} $Å^{-2}$ s^{-1} and (b) 167 e^{-} $Å^{-2}$ s^{-1}.

Figure S4 and S5 Length and width histograms of bulk experiments in acidic, basic and reductive conditions

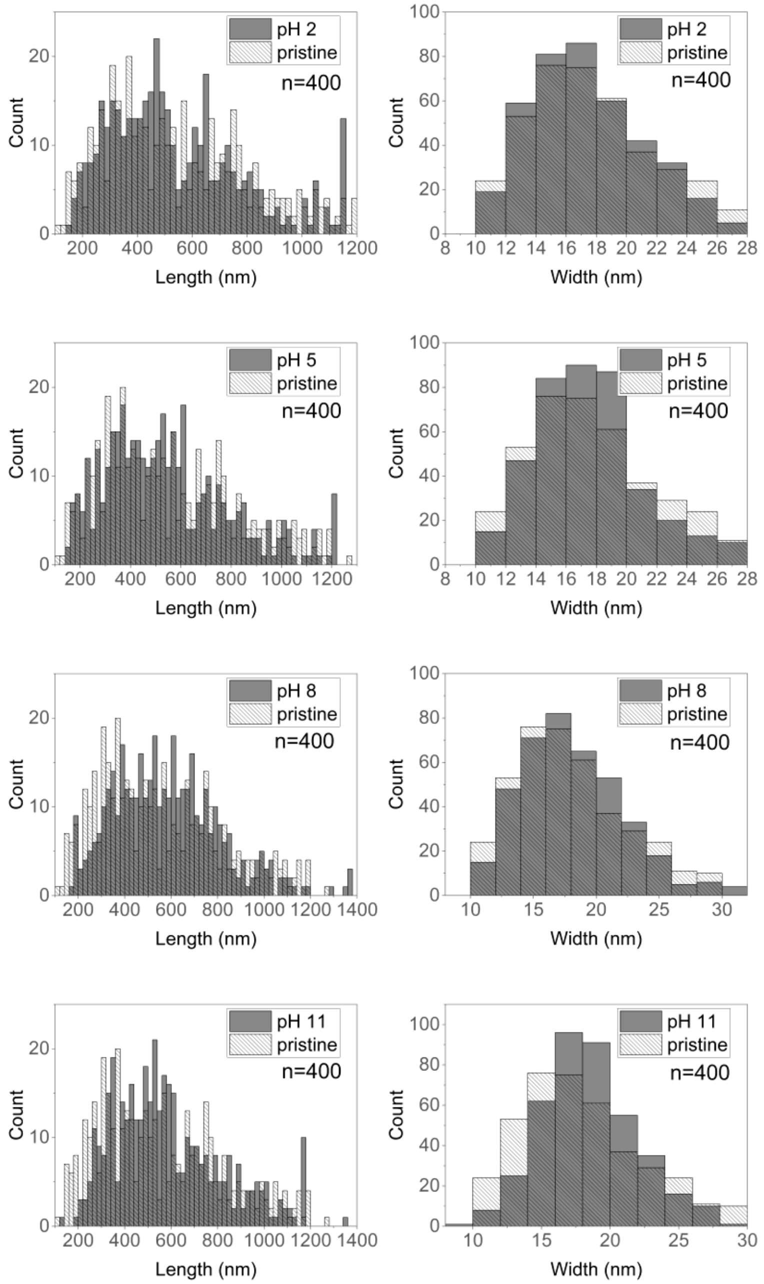

Figure S4: Frequency distribution of length and width dimensions of particles exposed to solutions of ultrapure water at pH 2, 5, 8, and 11 for 24 h

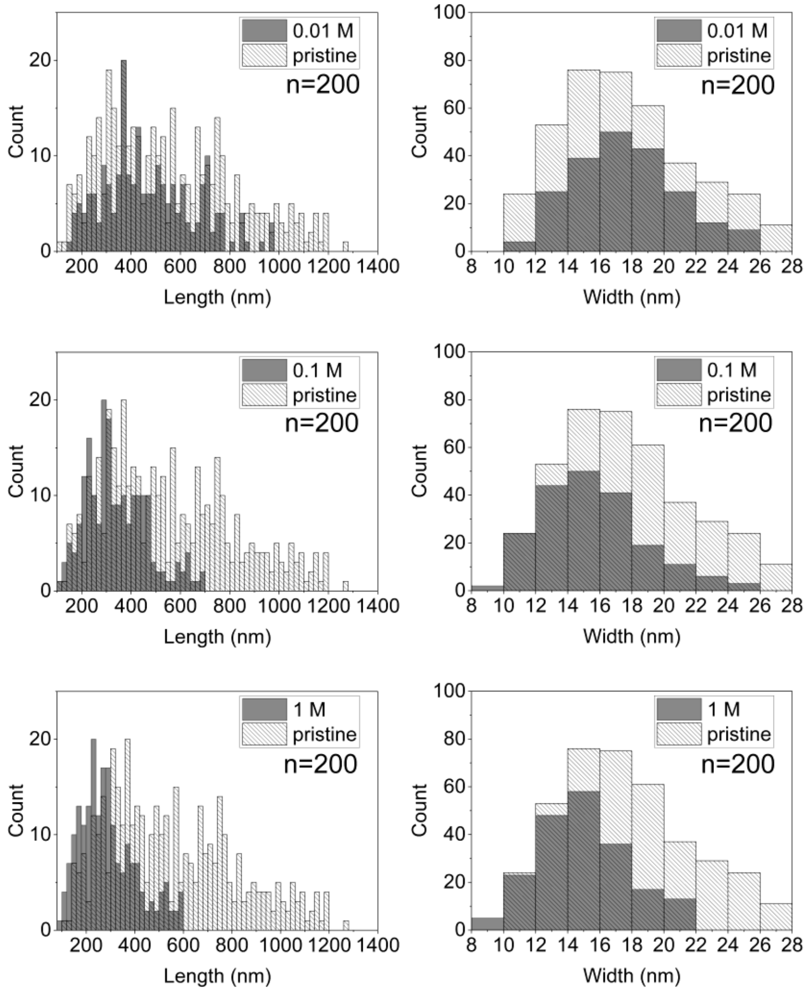

Figure S5: Frequency distribution of length and width dimensions of particles exposed to solutions at pH 5 in presence of 1, 0.1, and 0.01 M of hydroxylamine in ultrapure water for 24 h (n=200-400)

Figure S6 Estimation of iron released after 100 seconds exposure to 167 e^{-} $Å^{-2}$ s^{-1}

According to the volume loss during electron beam induced dissolution at high electron fluxes, it is possible to estimate an amount of iron released in the surrounding medium. To do so, geometric approximations were made from the 2D projection images and FIB-SEM transversal section. The first one concerns the layout of the particle settling on the substrate surface. As seen in Figure S6a, the isolated measured particles were settled on their longest crystalline face on the substrate. Then, TEM only provides the 2D projection of the object of interest on a projection screen or on the CCD detector. Therefore, it only gives access to projected length and width but not the equivalent of height necessary to calculate a volume of particles. To overcome this, TEM analysis of a FIB cross section of the particles aimed at accessing that missing dimension. Figure S6a present the representative perpendicular cut of goethite particle. The transversal section of the particles is a parallelogram. Therefore, the volume of each particle can be calculated by multiplying the area of the transversal section to the length of the particles. The length is directly estimated by the 2D projection of the particle length on the images, however the calculation of the transversal section is more complex. As detailed in Figure S6b, the area of a parallelogram is calculated by the edge times the height of the parallelogram. In our case, we can only access the 2D projection of particle width *w*. However, from this measure it is possible to obtain an approximated area of the transversal section. First the 2D projection of width can be related to the actual longer diagonal of the section though Pythagorean theorem, then, the height *h* in the goethite present particle can be approximated to half of the longer diagonal of the section. Using these parameters, the

section area is estimated as $A = w \cdot w/4$. The estimation of area through this calculation was confirmed by the error estimation of the actual area measures on the particle section. The calculation error when measuring area though the approximation was between 3-12% when compared to the theory of a perfect parallelogram area.

Through this method, we estimated the amount of iron released in the liquid cell image Figure 5b after 100 seconds of irradiation to 2.8 10^{-18} mol of iron released to the surrounding medium.

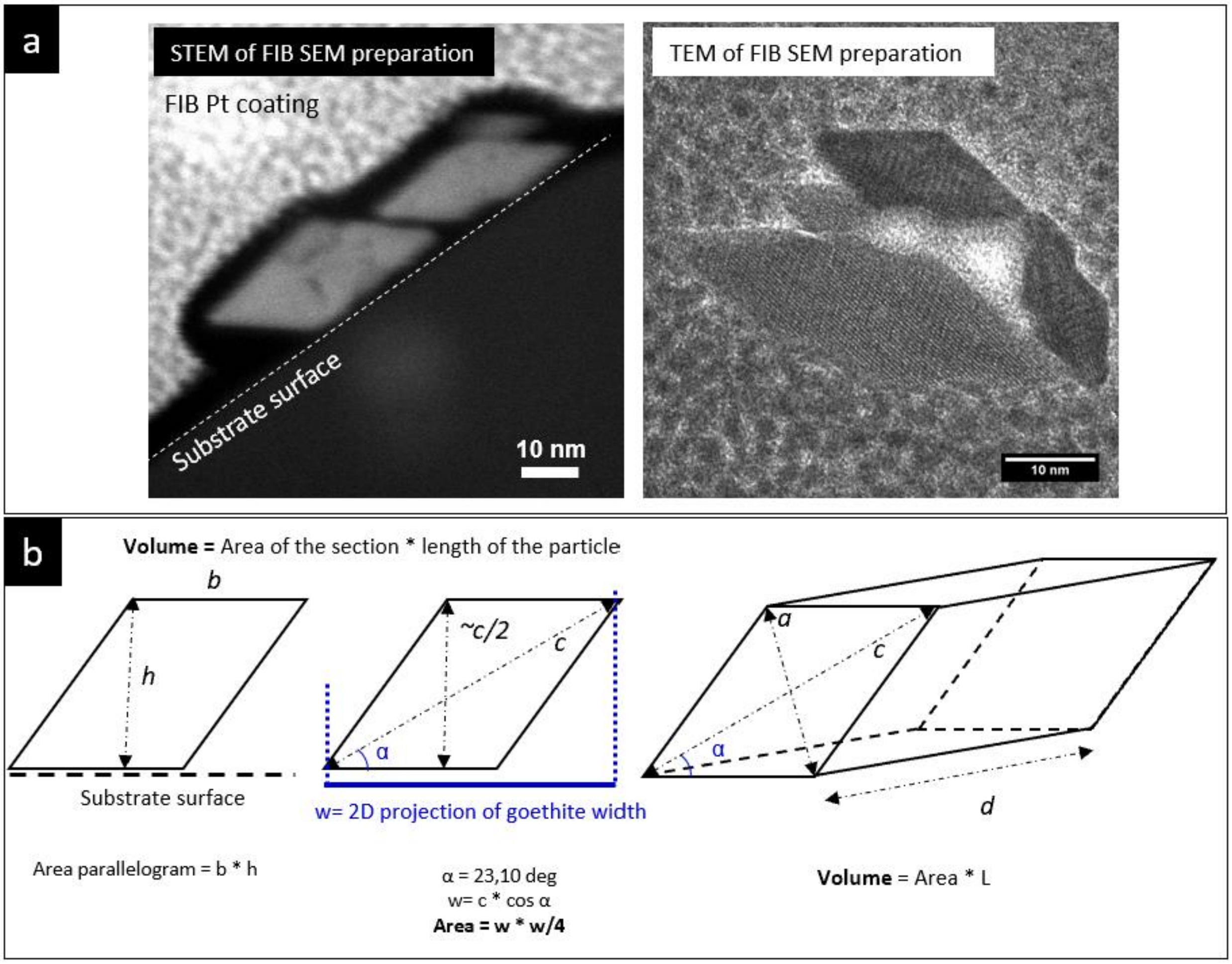

Figure S6: FIB-SEM transection of goethite nanoparticles observed in (a) STEM and (b) TEM and (c) scheme of geometric approximations of the section surface estimation

Table S3 Goethite dissolution rates derived for bulk and *in situ* experiments and literature values

Conditions	g Fe h^{-1} m^{-2}	References (main article)
in situ 21 e^{-} $Å^{-2}$ s^{-1}	7.61E-02	
in situ 167 e^{-} $Å^{-2}$ s^{-1}	1.64E-01	
bulk pH 2	1.18E-05	
bulk 1M NH_2OH	3.64E-04	

0.5 M HCl	1.00E-06	[26]
0.5 M HCl	1.00E-06	[39] acicular group1
0.5 M HCl	1.00E-06	[40]
30 mM sodium dithionite	1.03E-04	[41]

Figure S7 & S8 Analysis of $\pi^*$and K_w* and simulated steady state concentrations

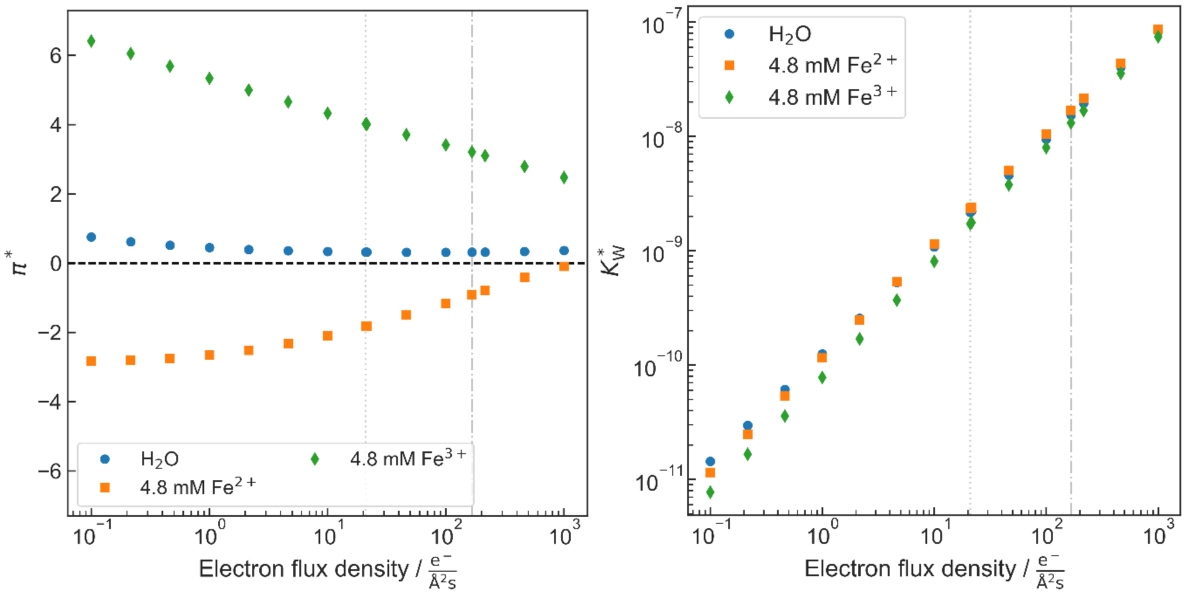

*Figure S7: Simulated π*and K_w* in presence of 4.8 mM of dissolved iron Fe^{2+} (orange squares) and Fe^{3+} (green diamonds). Pure water (blue circles) is plotted for comparison. Dotted and dashed lines represent the experimental conditions during this study.*

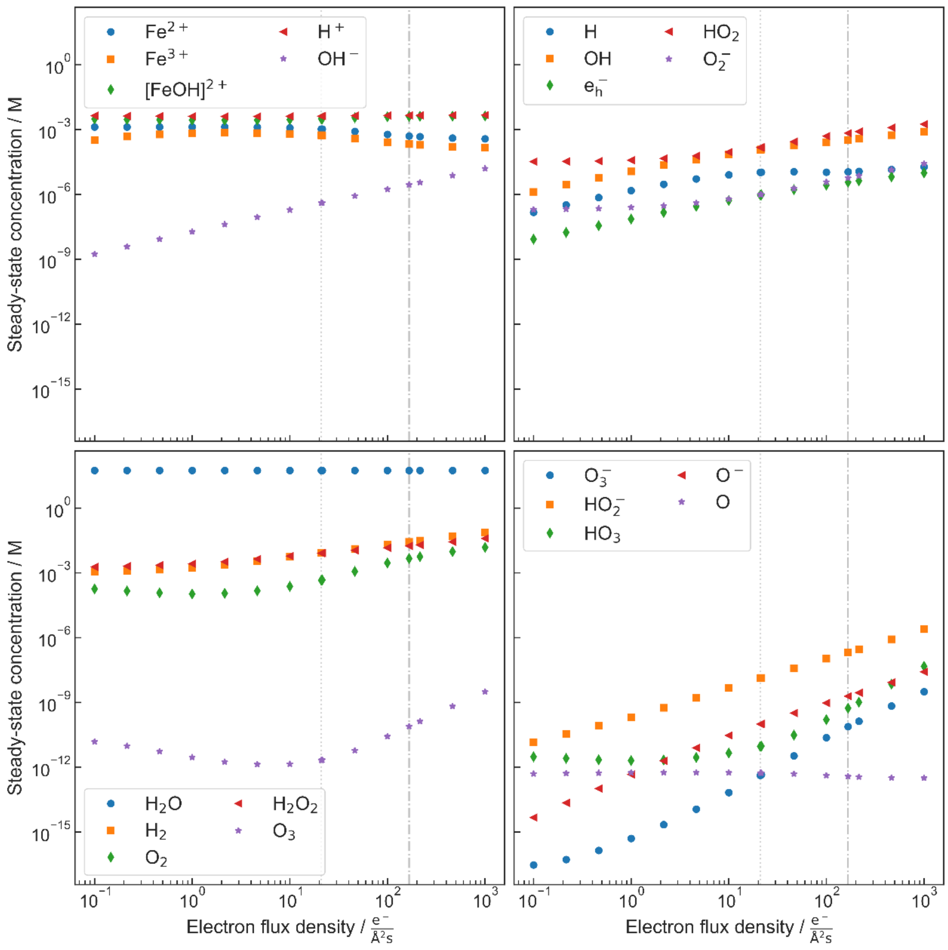

Figure S8: Simulated steady state concentrations of radical, ionic and molecular species for aqueous solutions containing 4.8 mM Fe^{3+}. Dotted and dashed lines represent respectively an electron flux density of 21 e^{-} $Å^{-2}$ s^{-1} and 167 e^{-} $Å^{-2}$ s^{-1}.

Supplementary references

[1] N. M. Schneider, M. M. Norton, B. J. Mendel, J. M. Grogan, F. M. Ross, H. H. Bau, *The Journal of Physical Chemistry C* **2014**, *118*, 22373.

[2] B. Fritsch, T. S. Zech, M. P. Bruns, A. Körner, S. Khadivianazar, M. Wu, N. Zargar Talebi, S. Virtanen, T. Unruh, M. P. M. Jank, E. Spiecker, A. Hutzler, *Advanced Science* **2022**, *9*, 2202803.

[3] W. P. Kwan, B. M. Voelker, *Environ. Sci. Technol.* **2002**, *36*, 1467.

[4] Z. Stuglik, Z. PawełZagórski, *Radiation Physics and Chemistry (1977)* **1981**, *17*, 229.

[5] J. D. Rush, B. H. J. Bielski, *The Journal of Physical Chemistry* **1985**, *89*, 5062.

[6] T. Loegager, J. Holcman, K. Sehested, T. Pedersen, *Inorg. Chem.* **1992**, *31*, 3523.

[7] M. Kelm, E. Bohnert, *A Kinetic Model for the Radiolysis of Chloride Brine, Its Sensitivity against Model Parameters and a Comparison with Experiments*, **2004**.

[8] C. D. Jonah, J. R. Miller, M. S. Matheson, *J. Phys. Chem.* **1977**, *81*, 1618.

[9] J. H. Baxendale, R. S. Dixon, D. A. Stott, *Trans. Faraday Soc.* **1968**, *64*, 2398.

[10] J. Lee, D. Nicholls, N. D. Browning, B. L. Mehdi, *Phys. Chem. Chem. Phys.* **2021**, *23*, 17766.

[11] C. M. Bethke, *Geochemical and Biogeochemical Reaction Modeling*, Cambridge University Press, **2007**.
